# ATM regulates NF-κB-dependent immediate-early genes via RelA Ser 276 phosphorylation coupled to CDK9 promoter recruitment

**DOI:** 10.1093/nar/gku529

**Published:** 2014-06-21

**Authors:** Ling Fang, Sanjeev Choudhary, Yingxin Zhao, Chukwudi B Edeh, Chunying Yang, Istvan Boldogh, Allan R. Brasier

**Affiliations:** 1 Department of Internal Medicine, University of Texas Medical Branch (UTMB), 301 University Blvd, Galveston, TX 77555 USA; 2 Department of Biochemistry and Molecular Biology, UTMB, Galveston, TX 77555, USA; 3 Sealy Center for Molecular Medicine, UTMB, 301 University Blvd, Galveston, TX 77555, USA; 4 Institute for Translational Sciences, UTMB, 301 University Blvd, Galveston, TX 77555, USA; 5 Department of Radiation Oncology, Houston Methodist Research Institute, Weill Cornell University, Houston, TX 77030, USA; 6 Department of Microbiology and Immunology, UTMB, 301 University Blvd, Galveston, TX 77555, USA

## Abstract

Ataxia-telangiectasia mutated (ATM), a member of the phosphatidylinositol 3 kinase-like kinase family, is a master regulator of the double strand DNA break-repair pathway after genotoxic stress. Here, we found ATM serves as an essential regulator of TNF-induced NF-kB pathway. We observed that TNF exposure of cells rapidly induced DNA double strand breaks and activates ATM. TNF-induced ROS promote nuclear IKKγ association with ubiquitin and its complex formation with ATM for nuclear export. Activated cytoplasmic ATM is involved in the selective recruitment of the E3-ubiquitin ligase β-TrCP to phospho-IκBα proteosomal degradation. Importantly, ATM binds and activates the catalytic subunit of protein kinase A (PKAc), ribosmal S6 kinase that controls RelA Ser 276 phosphorylation. In ATM knockdown cells, TNF-induced RelA Ser 276 phosphorylation is significantly decreased. We further observed decreased binding and recruitment of the transcriptional elongation complex containing cyclin dependent kinase-9 (CDK9; a kinase necessary for triggering transcriptional elongation) to promoters of NF-κB-dependent immediate-early cytokine genes, in ATM knockdown cells. We conclude that ATM is a nuclear damage-response signal modulator of TNF-induced NF-κB activation that plays a key scaffolding role in IκBα degradation and RelA Ser 276 phosphorylation. Our study provides a mechanistic explanation of decreased innate immune response associated with A-T mutation.

## INTRODUCTION

Nuclear factor-κB (NF-κB) is a major transcription factor that plays a central role in triggering the innate and adaptive immune response (1,2). In response to extracellular ligands or pathogen associated molecular patterns (PAMPs), activated death domain-containing receptors assemble submembranous signaling complexes to initiate signaling events for NF-κB activation (1,3). The central mechanism of NF-κB regulation is the signal dependent post-translational modifications of the IκB kinase (IKK) complex leading to phosphorylation and subsequent proteosomal degradation of a family of cytoplasmic NF-κB inhibitors, the IκBs (4). This process releases the sequestered, inactive RelA·p50 heterodimer. Current work has shown that RelA release from IκB complex is necessary, but not sufficient, for activation of the innate response. An overlapping ROS signaling pathway converging on members of the Ribosomal S6 Kinase family is involved in activating phosphorylation of RelA at serine (Ser) residue 276, an event coupled to its acetylation and co-activator recruitment (5). The fully activated RelA complex mediates phosphorylation of RNA polymerase II leading to transcriptional elongation and induction of gene expression of a subset of immediate-early cytokine genes (6). In this manner, a cell transduces external environmental signaling into regulated patterns of gene expression.

In a mechanism separate from PAMP signaling, endogenous danger-associated molecular patterns (DAMPs) trigger innate pathways via intracellular signaling independently of plasma membrane associated receptors (7). One example is the DNA damage response, where double-stranded DNA breaks (DSBs) induce a coordinated set of signaling events that activates a nuclear kinase ataxia-telangiectasia mutated (ATM), a member of the phosphotidylinositol 3 kinase-like kinase family of Ser/Thr-protein kinases. In addition to coordinating DNA damage repair by activating the homologous recombination pathway, active ATM undergoes cytosolic translocation to directly activate IKK and the NF-κB pathway. In this process, activated ATM binds to and phosphorylates Ser 85 of IKKγ, stimulating its ubiquitin-dependent nuclear export (8). The cytosolic ATM·IKKγ complex then activates the IKK complex and subsequent NF-κB-dependent gene expression, via a nuclear-cytoplasmic (‘inside-out’) signaling pathway. The role of ATM in contributing to receptor-initiated (‘outside-in’) signaling has not yet been described.

Recently, it has been appreciated that ATM regulates cellular functions unrelated to the DNA repair pathway that encompasses a vast array of cellular functions such as regulation of redox and energy status (9). Several studies have linked ATM deficiency to increased oxidative stress in cells resulting in various metabolic diseases, oncogenesis and neurodegeneration (10–12). Conversely, oxidative stress itself can induce ATM activation via oxidation of its Cys 2991 resulting into a disulfide-crosslinked ATM dimer (13). A-T patients expressing mutated ATM develop insulin resistance and type-2 diabetes (14). In this context, ATM is not only required for optimal activation of the Ser/Thr kinase AKT after insulin treatment but also for the expression of insulin growth factor-1 receptor. These reports clearly suggest a more versatile role of ATM in cellular metabolic processes independently of DSB repair.

In our high-throughput short interfering (siRNA) screening of the human kinome, we identified ATM as one of the novel kinases regulating TNF-induced canonical NF-κB pathway activation (15). These intriguing results suggested that intracellular danger signals modify the conventional outside-in signaling pathways triggered by the TNF receptor super family. In this study, we further investigated the role of ATM in proinflammatory chemokine/cytokine expression and provided a potential mechanism for how ATM mediates NF-κB activation after TNF treatment. We observed that TNF-induced ROS generation is essential for IKKγ association with polyubiquitin, a step required for its binding to activated (Ser 276 phosphorylated) ATM followed by nuclear export. Cytosolic ATM facilitates optimal IκBα proteosomal degradation by regulating recruitment of β-TrCP in the IκBα degradation complex. Lastly, cytosolic ATM regulates RelA Ser 276 phosphorylation, a post-translational modification (PTM) that controls a subset of the inflammatory NF-κB-dependent gene network. Our findings provide a mechanistic link for how the genetic syndrome produced by ATM is linked to defects in innate and adaptive immunity.

## MATERIALS AND METHODS

### Cell cultures and reagents

Human A549 pulmonary type II epithelial cells (American Type Culture Collection) were cultured in F-12K medium (Gibco, Invitrogen) with 10% fetal bovine serum, 1.0-mM sodium pyruvate, penicillin (100 U/ml) and streptomycin (100 g/ml) at 37°C in a 5% CO_2_ incubator. ATM^+/+^ and ATM^−/−^ MEFs are gifts from Dr Zhao-hui Wu and were cultured in Dulbecco's modified Eagle's medium (Gibco, Invitrogen) with 10% fetal bovine serum, 0.1-mM nonessential amino acids, 1.0-mM sodium pyruvate, penicillin (100 U/ml) and streptomycin (100 g/ml) at 37°C in a 5% CO_2_ incubator.

ATM, pATM and pRelA (Ser 276) antibodies (Ab Cat. No. 3037) were purchased from Cell Signaling Technology (Beverly, MA, USA). Lamin B Ab was purchased from EMD Millipore (Darmstadt, Germany). b-tubulin, IKKg, IkBa, pIkBa, b-TrCP, RelA, PKAc and CDK9 antibodies were purchased from Santa Cruz Biotechnology (Santa Cruz, CA, USA). NF-kB2/p100, pIKKb and pPolII antibodies were purchased from Abcam (Cambridge, UK).

### Nuclear and cytoplasmic extraction

Cytoplasmic and nuclear extracts were prepared as described earlier (16). Briefly, cells were harvested in PBS and centrifuged to collect pellets. Pellets were resuspended in double cell volume of solution A (50 mM HEPES (pH 7.4), 10 mM KCl, 1 mM EDTA, 1 mM EGTA, 1 mM dithiothreitol (DTT), 0.1 μg/ml phenylmethylsulfonyl fluoride, 1 μg/ml pepstatin A, 1 μg/ml leupeptin, 10 μg/ml soybean trypsin inhibitor, 10 μg/ml aprotinin and 0.5% IGEPAL-630) and centrifuged to obtain the supernatants as cytoplasmic fraction. The nuclear pellets were purified on sucrose cushions (buffer A with 1 M sucrose). After high salt extraction in buffer C (10% glycerol, 50 mM HEPES (pH 7.4), 400 mM KCl, 1 mM EDTA, 1 mM EGTA, 1 mM DTT, 0.1 μg/ml phenylmethylsulfonyl fluoride, 1 μg/ml pepstatin A, 1 μg/ml leupeptin, 10 μg/ml soybean trypsin inhibitor and 10 g/ml aprotinin), the nuclei were centrifuged at 12 000g at 4°C for 20 min. The supernatants were saved as NE. The protein concentrations were measured by Bradford protein assay (Protein Reagent, Bio-Rad).

### Western immunoblots

After SDS-PAGE electrophoresis, proteins were transferred to PVDF membrane and blocked in 5% milk/TBST buffer for 1 h. The membranes were incubated with specific primary antibody for overnight at 4°C followed by washing with TBST and incubation with secondary antibody for 1 h. Membranes were exposed by ECL western blotting solution (Amersham) and the films were developed by Kodak machine.

### Protein–protein interaction by co-immunoprecipitation

Cellular extracts (nuclear, cytoplasmic or whole cell extracts prepared in RIPA buffer) were precleared by incubating with protein A sepharose CL-4B (Sigma) beads for 45 min at 4°C. One milligram protein from precleared samples were incubated with 5 μg of specific antibody for overnight at 4°C. Immune complexes were captured by addition of protein A sepharose CL-4B (Sigma) for 2 h at 4°C. Following incubation, the beads were washed four times with TBS-T (50 mM Tris–HCl, pH 7.4, 150 mM NaCl, 5 mM EDTA and 0.05% Triton-x100) followed by the extraction of the bound protein boiling the beads in SDS-PAGE buffer containing b-mercaptoethanol for 5 min. The bound proteins were identified by performing western blot using specific antibodies.

### PKAc activity assay

Cells were lysed in a buffer containing 20 mM Tris–HCl, pH 7.5, 150 mM NaCl, 5 mM EDTA, pH 8.0, 1 mM PMSF, 10 μM leupeptin and 10 μM aprotinin. The cell suspension was sonicated for 30 s × 3, centrifuged and supernatant was collected. PKAc activity was measured in the supernatant using PKA-specific peptide (LRRASLG) according to the supplier's recommendations (PepTag assay, Promega, Madison, WI, USA). Phosphorylated and nonphosphorylated peptides were separated by electrophoresis on a 0.8% agarose gel and quantitated by measuring absorbance at 570 nm. Data were presented as percentage of absorbance relative to 0 h.

### q-RT-PCR

Total cellular RNA was extracted by Tri Reagent (Sigma-Aldrich). cDNA was synthesized from 1 μg of RNA using iScript cDNA Synthesis Kit (Bio-Rad). Three μl of cDNA products was amplified in 20 μl reaction volume containing 10 μl iQ SYBR Green Super Mix (Bio-Rad) and 400 nM primer mix. All reactions were processed in MyiQ Single-Color Real-Time PCR Detection System (Bio-Rad) and results were analyzed by IQ5 program (Bio-Rad).

### Chromatin immunoprecipitation

Two-step cross-link chromatin immunoprecipitation (ChIP) was performed as described previously (17). Briefly, A549 cells were washed twice with PBS and incubated with disuccinimidyl glutarate (2 mM for 45 min) for protein–protein cross-linking. Subsequently, protein–chromatin cross-linking was performed by incubating the cells with 1% formaldehyde for 30 min. Equal amounts of sheared chromatin were immunoprecipitated overnight with 4 ug of the indicated antibody in ChIP dilution buffer at 4°C. Immunoprecipitates were collected with 40 ul protein A magnetic beads (Invitrogen), washed and associated chromatin was eluted in 250 ul elution buffer for 15 min at room temperature. Samples were de-cross-linked in 0.2M NaCl at 65°C for 2–4 h. The precipitated DNA was phenol-chloroform extracted, precipitated with 100% ethanol, and dried. The promoter-specific primers used for quantitative PCR of each gene are as follows: Gro-β: forward: 5′- TCGCCTTCCTTCCGAACTC-3′, reverse: 5′- CGAACCCCTTTTATGCATGGT-3′; IκBα: forward: 5′- GACGACCCCAATTCAAATCG-3′, reverse: 5′- TCAGGCTCGGGGAATTTCC-3′; IL-8: forward: 5′- AGGTTTGCCCTGAGGGGATG-3′, reverse: 5′-GGAGTGCTCCGGTGGCTTTT-3′.

### Stable isotope dilution (SID)-selected Reaction Monitoring (SRM)-mass spectrometry (MS) assays

The SRM assays for total RelA, IKKγ and phospho-Ser 536 RelA were described previously (18). The SRM assay for phospho-Ser 276 RelA was developed using a workflow described previously (18). The signature peptides and MS parameters of each SRM assay are listed in Table 1. Stable isotope standard (SIS) peptides were chemically synthesized incorporating isotopically labeled [13C615N4] Arginine or [13C615N4] Lysine to a 99% isotopic enrichment (Thermo Scientific).

**Table 1.** SRM parameters of SRM assays of targeted proteins and post-translational modifications

Gene name	Swissprot number	Sequence	Q1 m/z	Q3 m/z	Ion type	CE (V)
RelA	Q04206	TPPYADPSLQAPVR	756.396	867.504	y8	27
			756.396	982.531	y9	27
			756.396	1053.568	y10	27
			756.396	1313.684	y12	30
IKKγ	Q9Y6K9	AQVTSLLGELQESQSR	873.455	976.469	y8	27
			873.455	1033.490	y9	27
			873.455	1146.574	y10	27
			873.455	1259.658	y11	27
			873.455	1346.690	y12	27
Phospho-Ser 536 RelA	Q04206	DFSS[Phosphoryl]IADMDFSALLSQISS	1057.455	521.256	y5	35
			1057.455	634.34	y6	35
			1057.455	747.424	y7	35
			1057.455	818.461	y8	35
			1057.455	905.493	y9	35
			1057.455	1008.467	Reporter	35
			1057.455	1052.562	y10	35
			1057.455	1167.589	y11	35
			1057.455	1298.629	y12	35
Phospho-Ser 536 RelA	Q04206	DFSS[Phosphoryl]IADM[Oxid]DFSALLSQISS	1065.453	521.256	y5	35
			1065.453	634.34	y6	35
			1065.453	747.424	y7	35
			1065.453	818.461	y8	35
			1065.453	905.493	y9	35
			1065.453	1016.464	Reporter	35
			1065.453	1052.562	y10	35
			1065.453	1167.589	y11	35
			1065.453	1314.624	y12	35
Phospho-Ser 276 RelA	Q04206	RPS[Phosphoryl]DR	355.653	306.664	Reporter	15
			355.653	457.144	y3	15
			355.653	554.197	y4	15
			355.653	710.298	y5	15

Masses listed are for the native forms of the peptides. Abbreviations: CE, collision energy; Q, quadropole.

Ubiquitin-associated IKKγ or RelA were immunoprecipitated from native extracts using anti-Ubiquitin or RelA Ab and immune complexes captured by protein A magnetic beads (Dynal Inc). For the measurement of the level of ubiquitin-conjugated IKKγ and phospho-Ser 276 RelA, the proteins on the beads were digested with trypsin as described previously (19). Briefly, the beads were washed with PBS for three times and then resuspended in 30 μl of 50 mM ammonium hydrogen carbonate (pH 7.8) and 20 μl of 0.1 μg/ μl of trypsin were added. The samples were mixed and trypsinized by gentle vortexing overnight at 37°C. After digestion, the supernatant was collected. The beads were washed with 50 μl of 50% acetonitrile (ACN) three times and the supernatant was pooled, and dried. The tryptic digests were then reconstituted in 30 μl of 5% formic acid-0.01% TFA for MS analysis. For the measurement of the level of phosphor-Ser 536 RelA, the protein on the beads were digested with Glu-C (Indianapolis, IN) as described previously (18). The peptides were extracted from the beads as described above and then reconstituted in 30 μl of 5% formic acid-0.01% TFA for MS analysis. An aliquot of 10 μl of diluted SIS peptides were added to each digest before LC-SRM-MS analysis.

LC-SRM-MS analysis was performed with a TSQ Vantage triple quadrupole mass spectrometer (Thermo Scientific, San Jose, CA) equipped with nanospray source (Thermo Scientific, San Jose, CA) as described before. The online desalting and chromatography were performed using an Eksigent NanoLC-2D HPLC system (AB SCIEX, Dublin, CA). An aliquot of 10 μl of each of tryptic digests were injected on a C18 peptide trap (Agilent, Santa Clara, CA), desalted with 0.1% formic acid at a flow rate of 2 μl/min for 45 min. Peptides were eluted from the trap and separated on a reversed phase nano-HPLC column (PicoFrit™, 75 μm x 10 cm; tip ID 15 μm) packed in house using Zorbax SB-C18 (5-μm diameter particles, Agilent, Santa Clara, CA). Separations were performed using a flow rate of 500 nl/min with a 20-min linear gradient from 2–40% mobile phase B (0.1% formic acid-90% ACN) in mobile phase A (0.1% formic acid), followed by 0.1-min gradient from 40–90% mobile phase B and 5-min 90% mobile phase B. The TSQ Vantage was operated in high-resolution SRM mode with Q1 and Q3 set to 0.2 and 0.7-Da Full Width Half Maximum. All acquisition methods used the following parameters: 1800 V ion spray voltage, a 275°C ion transferring tube temperature, a collision-activated dissociation pressure at 1.5 mTorr and the S-lens voltage used the values in S-lens table generated during MS calibration. All SRM data were manually inspected to ensure peak detection and accurate integration. The chromatographic retention time and the relative product ion intensities of the analyte peptides were compared to those of the SIS peptides. The variation of the retention time between the analyte peptides and their SIS counterparts were within 0.05 min, and no significant difference in the relative product ion intensities of the analyte peptides and SIS peptides were observed. The peak area in the extract ion chromatography of the native and SIS version of each signature peptide were integrated using Xcalibur® 2.1. The default values for noise percentage and base-line subtraction window were used. The ratio between the peak area of native and SIS version of each peptide were calculated.

### Comet assays

DNA double-strand breaks were quantitated by a Comet assay (Trevigen, Inc, Gaithersburg, MD) as we described previously (20). Briefly, cells were suspended in 0.6% low melting-point agarose. The cell/agarose suspension (80 μl) was applied onto pre-coated microscope slides (Trevigen Inc). Slides with solidified agarose were placed in lysis buffer (2.5 M NaCl, 100 mM EDTA, 10 mM Trish-HCl (pH 10), 1% sodium sarcosinate and 1% Triton X-100) for 24 h. Slides were placed in pre-cooled (4°C) electrophoresis tanks and electrophoresis was carried out at pH 7.8 in a buffer provided by the manufacturer (Trevigen Inc). After electrophoresis at 1.25 V per cm) at 4°C, preparations were air-dried and DNA stained by SYBER green. Comets were analyzed by using the Comet Assay IV v4.2 system (Perceptive Instruments, Suffolk, UK) image analysis software (4). The fold change in tail comet moments are expressed as the mean (± SD) from three parallel experiments.

### ShRNA-mediated ATM knockdown in A549 and HeLa cells

ATM and control shRNA are gifts from Dr Sankar Mitra (Methodist hospital, Houston, TX). Plasmids were transfected to A549 and HeLa cells using FuGENE HD kit (Promega, Madison, WI). Five μg plasmid was mixed with 15 μl of FuGENE HD Transfection Reagent for 5 min. Afterward, the mixture was added to a 10-cm cell plate and mixed gently. Forty eight hours later, cells were selected in puromycin (6 μg/ml) for 2 weeks.

## RESULTS

### TNF-induced ATM nuclear export requires IKKγ ubiquitination.

**Figure 1.**
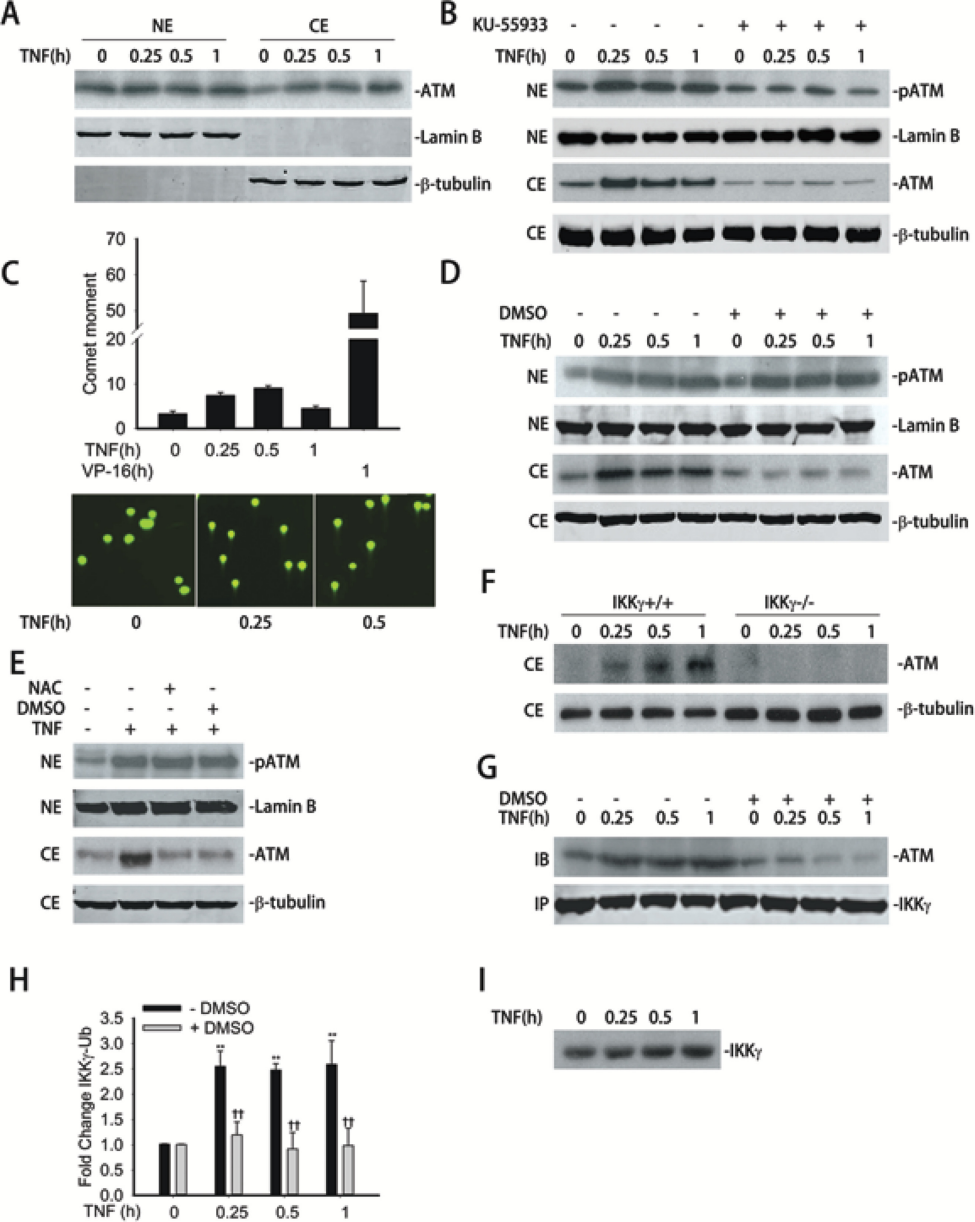
TNF-induced ATM activation and nuclear export. (**A**) A549 cells were treated with TNF (30 ng/ml) for the indicated time. Equal amounts of nuclear extract (NE) and cytoplasmic extract (CE) were analyzed by WBs to detect the level of ATM in both NE and CE. Lamin B and β-tubulin were also detected as internal control for NE and CE, respectively. (**B**) A549 cells were treated with TNF (30 ng/ml) for the indicated time with or without the pretreatment of KU-55933 (10 μM, 1 h). Equal amounts of NE and CE were analyzed by WBs to detect the level of pATM and ATM, respectively. Lamin B and β-tubulin were also detected as internal control for NE and CE, respectively. (**C**) Top panel. A549 cells were treated with TNF (30 ng/ml) or VP-16 (10 μM) for the indicated time and then assayed by Neutral Comet assay. 100 cells from each time interval were quantitated. Bottom panel. Representative images of comet moments. (**D**) A549 cells were treated with TNF (30 ng/ml) for the indicated time with or without the pretreatment of DMSO (2% in vol, 0.5 h). Equal amounts of NE and CE were analyzed by WBs to detect the level of pATM and ATM, respectively. Lamin B and β-tubulin were also detected as internal control for NE and CE, respectively. (**E**) A549 cells were treated with TNF (30 ng/ml) for 1 h with or without DMSO (2% in vol, 0.5 h) or NAC (15 mM, 1 h) pretreatment. Equal amounts of NE and CE were extracted and analyzed by WBs to detect the level of pATM and ATM, respectively. Lamin B and β-tubulin were also detected as internal control for NE and CE, respectively. (**F**) IKKγ^+/+^ and IKKγ^−/−^ MEFs were treated with TNF (30 ng/ml) for the indicated time. Equal amounts of CE were analyzed by WB to detect the level of ATM. β-tubulin were detected as internal control. (**G**) A549 cells were treated with TNF (30 ng/ml) for the indicated time with or without the pretreatment of DMSO (2% in vol, 0.5 h). Equal amount of NE were immunoprecipitated by anti-IKKγ Ab. Interacting ATM were measured by WBs. (**H**) A549 cells were treated with TNF (30 ng/ml) for the indicated time with or without the pretreatment of DMSO (2% in vol, 0.5 h). Equal amount of NE were immunoprecipitated by Ubiquitin Ab and subjected to SID-SRM-MS analysis of IKKγ protein level. All of the values are presented as the ratios of native to SIS peptides. (**I**) A549 cells were treated with TNF (30 ng/ml) for the indicated times, Equal amount of NE were immunoprecipitated by anti-IKKγ Ab and assayed by western blot using anti-IKKγ Ab. * Significantly different from TNF (0 h)-treated samples, *P* < 0.05;** Significantly different from TNF (0 h)-treated samples, *P* < 0.01; ^†^ Significantly different from ATM^+/+^ samples, *P* < 0.05; ^††^ Significantly different from ATM^+/+^ samples, *P* < 0.01.

In unstimulated cells, ATM resides in the nucleus as an inactive form. Following DNA double-strand breaks (DSBs), it undergoes autophosphorylation at Ser 1981 (21). ATM activation has been reported to be coupled with nuclear export in the DSB-induced DNA repair pathway (8). Therefore, we investigated whether a similar phenomenon happens upon TNF stimulation. Nuclear (NE) and cytoplasmic extract (CE) were prepared from cells stimulated with TNF and assayed by Western immunoblot. First, Lamin B and β-tubulin were detected to characterize subcellular enrichment. Here, we observed that although the nuclear fractions stained strongly with Lamin B, the cytosolic fractions did not, indicating that CEs were largely devoid of nuclear contamination (Figure 1A). We next examined the distribution of ATM using anti-ATM Ab. Under unstimulated conditions, an ∼350 kDa ATM band is primarily located in the nucleus and present in lower abundance in the cytosol. By contrast, after 0.25 h of TNF stimulation, ATM is detected in the cytoplasmic fraction where it continues to accumulate until 1 h of stimulation (Figure 1A). From this experiment, we concluded that TNF induces ATM nuclear-to-cytoplasmic transport.

In order to determine whether ATM kinase activity is required for TNF-induced nuclear export, we utilized the specific ATM kinase inhibitor, KU-55933 (22). A549 cells were pre-treated with the KU-55933 (10 μM, 1 h) before TNF exposure. NE and CE were prepared and assayed by western immunoblot. In the NE, phospho-Ser 1981 ATM (pATM) was observed in the absence of stimulation, whose abundance was induced within 0.25 h of TNF stimulation (Figure 1B). By contrast, a lower abundance of pATM was seen in cells treated with KU-55933 only, and no TNF-inducible accumulation was observed (Figure 1B, top right panel). Correspondingly, the appearance of ATM in the CE was detected upon TNF stimulation while KU-55933 pretreatment completely blocked its cytoplasmic accumulation (Figure 1B, third panel). Our observations that KU-55933 pretreatment completely blocks TNF-induced pATM formation as well as cytoplasmic accumulation suggest that ATM kinase activity is prerequisite for its TNF-inducible nuclear export.

We have reported a significant and transient induction of ROS generation after TNF treatment as measured by H2DCF oxidation, increased in 8-oxoguanine (8-oxoG) DNA lesions, and by protein carbonylation (5). Although elevated ROS and 8-oxoG formation is usually associated with ssDNA breaks, we evaluated the possibility whether TNF treatment is sufficient to induce DNA double strand breaks (DSBs) and provide a potential mechanism of TNF-induced ATM activation and nuclear export. A549 cells were TNF treated for various times intervals followed by Neutral Comet assay, an assay that specifically detects DSBs (23). Etoposide (VP-16), which is a traditional drug to induce significant DSBs, is used as a positive control. A significant, but transient, 2-fold increase in DSB formation was observed after 0.25 h of TNF exposure that further increased to 2.5-fold at 0.5 h before declining to untreated levels by the end of 1 h exposure (Figure 1C, top panel). On the other hand, VP-16 induced much higher (∼42-fold) level of DSBs than that induced by TNF. Representative microscopic images of comet moment after TNF stimulation is shown in Figure 1C (bottom panel). These data suggest that TNF induces DSBs, albeit at much lower levels than that induced by VP-16.

To ascertain whether TNF-induced ROS generation is essential for pATM formation and its subsequent nuclear export, A549 cells were pretreated with the free radical scavenger, DMSO (2% for 0.5 h), at concentrations that we have previously shown to be sufficient to block TNF-induced ROS formation without apparent cytotoxicity (5). CE and NE were prepared from a TNF time course in the presence or absence of DMSO. In NE, we observed an induction of pATM upon TNF stimulation, but surprisingly, DMSO pretreatment did not affect pATM formation (Figure 1D, top panel). By contrast, DMSO pretreatment blocked the rapid kinetics of cytoplasmic accumulation of ATM. Here, we noted that in the absence of DMSO, cytoplasmic ATM is detectable within 0.25 h, whereas in the presence of DMSO, cytoplasmic ATM is markedly reduced (Figure 1D, middle panel). To further validate our surprising findings, we utilized another chemically unrelated ROS inhibitor, NAC (N-acetyl cysteine) (24), to see whether pretreatment affects ATM phosphorylation and ATM export. Similar to the results of the DMSO pretreatment, NAC pretreatment blocked ATM export without affecting pATM formation (Figure 1E). This result suggests that ROS affects ATM cytoplasmic translocation at a step downstream of pATM formation (25).

Previous work showed that DSB-induced ATM export is IKKγ-dependent, where nuclear complex formation of pATM and IKKγ is prerequisite for NF-κB activation. In this mechanism, activated ATM phosphorylates IKKγ on Ser 85 to promote its ubiquitination (Ub) and nuclear export (8). Cytoplasmic export of the ATM·Ub-IKKγ complex allows it to associate with and activate IKK, the rate-limiting step in NF-κB liberation from cytoplasmic stores. Therefore, we assessed if TNF-induced ATM nuclear export also is IKKγ-dependent. For this purpose, IKKγ^+/+^ and IKKγ^−/−^ MEFs were stimulated by TNF for various length times, CE were prepared and immunoblotted with anti-ATM antibody (Ab). Although ATM translocation into the cytosol was observed in IKKγ^+/+^ MEFs, ATM export was completely blocked in IKKγ^−/−^ cells suggesting that IKKγ is essential for TNF-induced nuclear export of ATM (Figure 1F).

Next, we evaluated the effect of ROS on ATM·IKKγ complex formation. Nuclear IKKγ was immunoprecipitated from TNF treated A549 cells in the absence or presence of DMSO pretreatment. Levels of IKK-associated ATM were quantitated in the immunoprecipitates by Western blot. We noted that TNF induced ATM·IKKγ complex formation in a kinetic that matches with pATM phosphorylation and nuclear export. However, a distinct pattern was observed with DMSO pretreatment. Here, the ATM·IKKγ association was observed in DMSO only treated cells, and upon TNF stimulation, the abundance of the ATM·IKKγ complex reduced over time (Figure 1G). These data suggest that TNF-inducible ATM·IKKγ interaction is ROS-dependent (Figure 1G). Previous studies have shown that IKKγ ubiquitination at Lys 285 is essential for ATM-NF-κB pathway (26). Therefore, to further understand the role of TNF-induced ROS on ATM·IKKγ binding and subsequent nuclear export, we assessed its effect on IKKγ ubiquitination. For this measurement, NEs from A549 cells stimulated with TNF with or without DMSO pretreatment were used. Ubiquitinated nuclear proteins were enriched by immunoprecipitation in native extracts using an ubiquitin-specific Ab. IKKγ was quantified by a quantitative, selected reaction monitoring-mass spectrometry (SID-SRM-MS) assay using a prototypic peptide that detects unmodified IKKγ. Within 0.25 h of TNF treatment, Ub-associated IKKγ levels increased by 2.5-fold over untreated cells and persisted for 1 h. However, DSMO pretreatment completely blocked Ub association with IKKγ (Figure 1H). Previous studies have shown that unanchored polyubiquitin chains existing in the cells play essential roles in innate cell signaling pathways (27,28). We therefore investigated whether the increased level of Ub associated with IKKγ comes from conjugated or unconjugated polyubiquitin. NEs were immunoprecipitated with anti-IKKγ Ab and the formation of ubiquitin adducts measured by western blot using anti-ubiquitin Ab. No adducts could be detected (data not shown). We additionally probed the western blot using anti-IKKγ Ab. Only a single IKKγ band was detected without any additional higher molecular weight species (Figure 1I). These data suggest that ROS induce IKKγ binding to free polyubiquitin chains, or the abundance of the covalently linked IKKγ is below the limit of detection of our assay. Overall, our data suggests that TNF-induced DNA DSBs activates ATM and nuclear export. Additionally our data suggest that TNF-induced ROS is required for ATM export through affecting Ub-IKKγ-complex formation and ATM association.

### Cytoplasmic ATM is essential for TNF-α-induced IκBα degradation through β-TrCP recruitment.

**Figure 2.**
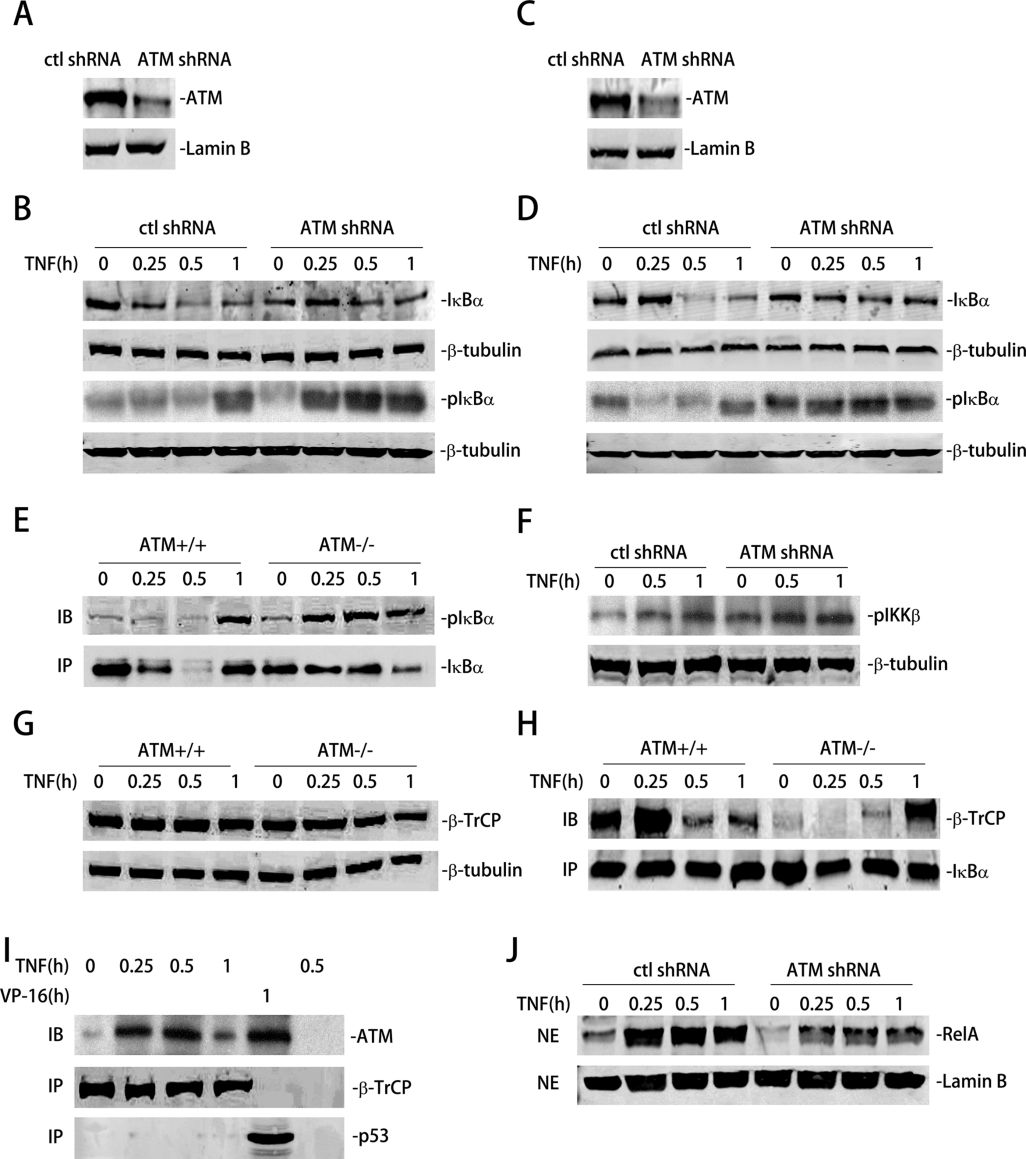
ATM is essential for IκBα degradation by recruiting β-TrCP. (**A**) A549 cells were transfected with ATM shRNA. 72 h later, equal amounts of NE were analyzed by WB to detect ATM. Lamin B were detected as internal control. (**B**) Control shRNA and ATM shRNA knockdown A549 cells were treated with TNF (30 ng/ml) for the indicated time. Equal amounts of CE were analyzed by WBs to detect the level of totalIκBα and pIκBα. β-tubulin were detected as internal control. (**C**) HeLa cells were transfected with ATM shRNA. 72 h later, equal amounts of NE were analyzed by WB to detect ATM. Lamin B were detected as internal control. (**D**) Control shRNA and ATM shRNA knockdown HeLa cells were treated with TNF (30 ng/ml) for the indicated time. Equal amounts of CE were analyzed by WBs to detect the level of total IκBα and pIκBα. β-tubulin were detected as internal control. (**E**) ATM^+/+^ and ATM^−/−^ MEFs were treated with TNF (30 ng/ml) for the indicated time. Equal amount of CE were immunoprecipitated by anti-IκBα Ab. pIκBα levek were measured by WBs. (**F**) Control shRNA and ATM shRNA transfected A549 cells were TNF treated (30 ng/ml) for the indicated times. Equal amounts of WCE were analyzed by Western blot using anti-pIKKβ Ab. β-tubulin was used as an internal control. (**G**) ATM^+/+^ and ATM^−/−^ MEFs were treated with TNF (30 ng/ml) for the indicated time. Equal amount of CE were were analyzed by WB to detect β-TrCP. β-tubulin were detected as internal control. (**H**) ATM^+/+^ and ATM^−/−^ MEFs were pre-treated with MG132 prior to treatment with TNF (30 ng/ml) for the indicated time. Equal amount of CE were immunoprecipitated by anti-IκBα Ab. Interacting β-TrCP were measured by WBs. (**I**) A549 cells were treated with TNF (30 ng/ml) or VP16 (10 μM) for the indicated time. Equal amount of CE were immunoprecipitated by anti-β-TrCP Ab (lane 1–4), anti-p53 Ab (lane 5) or rabbit IgG (lane 6). Interacting ATM were measured by WBs. (**J**) Control shRNA and ATM shRNA-transfected A549 cells were treated with TNF (30 ng/ml) for the indicated times. Equal amounts of NE were analyzed by western to detect the level of RelA. Lamin B was detected as internal control.

In our earlier report, we observed a delay in TNF-inducible IκBα proteolysis and decreased RelA nuclear translocation in ATM^−/−^ MEFs, suggesting a novel role of cytoplasmic ATM in facilitating IκBα degradation (15). Here, we further extend this observation and sought to elucidate the mechanism by which ATM alters IκBα degradation. We first sought to reproduce the findings using shRNA-mediated knockdown of ATM in human lung epithelial cells (A549). ATM-directed shRNA decreased ATM expression by 80% (Figure 2A). TNF-induced IκBα degradation was measured in CEs using western immunoblot. In control A549 cells, there was no marked IκBα degradation after 0.25 h; however, a rapid disappearance was observed 0.5 h after TNF treatment followed by IκBα resynthesis within 1 h. By contrast, in the ATM-depleted cells IκBα degradation was significantly delayed, not being apparent until after 1 h of TNF treatment (Figure 2B). Similar findings were observed in experiments using shRNA mediated ATM knockdown in human cervical cancer epithelial (Hela) cells. IκBα was not affected after 0.25 h, but rapidly degraded 0.5 h in control shRNA transfected cells. This proteolysis was blocked after ATM knockdown (Figure 2C and D). Together we conclude that ATM affects TNF-induced IκBα degradation.

Previous work has shown that TNFα activates the IKK complex, a rate-limiting kinase responsible for IκBα phosphorylation at Ser residues 32 and 36 in its NH2 terminal regulatory domain. Phospho-Ser 32/36 IκBα (pIκBα) is specifically bound by the β-TrCP ubiquitin ligase, triggering recruitment to the 26S proteasome and its degradation (29,30). We first investigated if ATM affects IκBα phosphorylation. pIκBα was detected in whole cell extracts (WCE) from time course of TNF stimulation in control or ATM knockdown A549 cells. In control shRNA transfected cells pIκBα were detectable in unstimulated cells and was depleted by 0.5 h, similar to the profiles for total IκBα degradation (Figure 2B). In contrast, pIκBα accumulates immediately after TNF stimulation in ATM shRNA transfected cells, and persists throughout the time course (Figure 2B). Similar findings were observed in shRNA mediated HeLa cells (Figure 2D). These results demonstrate that pIκBα is stabilized in ATM-deficient cells.

To confirm the effects of ATM on stabilizing pIκBα levels, WCEs from a time course of TNF stimulation in ATM^+/+^ and ATM^−/−^ MEFs were prepared and total IκBα was immunoprecipitated. The level of pIκBα was then detected by western immunoblot. ATM^+/+^ MEFs showed a low level of pIκBα in untreated cells. By contrast, there was an accumulation of pIκBα in ATM^−/−^ MEFs (Figure 2E). Although the level of pIκBα was difficult to detect in ATM^+/+^ cells due to its proteolysis, the abundance of pIκBα in ATM^−/−^ MEFs showed marked accumulation compared to that of ATM^+/+^ MEFs (Figure 2E). To further extend these findings, the same experiment was done using control shRNA and ATM shRNA transfected A549 cells. A similar result was observed (Supplementary Figure S1). These data further suggest that pIκBα stabilization is a consistent finding for ATM^−/−^ MEFs and ATM knockdown human cell lines.

To exclude the potential role of ATM in upstream effects on the TNF signaling pathway, we examined pIKKβ activation, the kinase directly upstream of IκBα (4). WCE from TNF stimulated control- and ATM knockdown A549 cells were assayed by western blot using anti-pIKKβ Ab. We observed indistinguishable induction of pIKKβ in both cell lines (Figure 2F). We therefore conclude that TNF-induced pIKKβ formation is ATM-independent.

Recruitment of β-TrCP to pIκBα is essential for its ubiquitination and proteolysis. One explanation could be that the differences in IκBα proteolysis were due to differences in β-TrCP expression. To address this possibility, CEs from TNF stimulated ATM^+/+^ and ATM^−/−^ MEFs were prepared and assayed for steady-state β-TrCP levels. We observed that the level of β-TrCP protein expression was identical in ATM^+/+^ and ATM^−/−^ MEFs (Figure 2G). Therefore, we assessed whether ATM influenced formation of the IκBα·β-TrCP complex. In this experiment, ATM^+/+^ and ATM^−/−^ MEFs pre-treated with MG132 (to block proteosomal activity) were stimulated with TNF. IκBα complexes were enriched by immunoprecipitation and β-TrCP level detected by immunoblot. In ATM^+/+^ cells, β-TrCP was engaged with IκBα at a low level in the absence of stimulation and transiently increased after 0.25 h of treatment (Figure 2H). A subsequent decline in the abundance of the complex was seen after 0.5 h. By contrast, the basal IκBα·β-TrCP interaction was significantly decreased in ATM^−/−^ MEFs, and was not detectable until after 1 h of stimulation (Figure 2H). To extend these findings, this experiment was also conducted in control- and ATM shRNA-transfected HeLa cells, where different kinetics of β-TrCP and IκBα association was observed (Supplementary Figure S2).

Since β-TrCP also targets NF-κB2/p100 for ubiquitin-mediated proteolysis, we asked whether ATM deficiency affects NF-κB2/p100 binding in response to noncanonical pathway activation (31). For this purpose, control- or ATM shRNA- transfected A549 cells were pre-treated with the proteasome inhibitor MG132 and subsequently TNF stimulated for 1 and 3 h, time points reported to induce p100 processing and non-canonical pathway activation in A549 cells (32). NF-κB2/p100-β-TrCP association was measured by NF-κB2/p100 western blot of β-TrCP immunoprecipitates. We detected induction of NF-κB2/p100-β-TrCP association at equivalent levels in both ATM replete and ATM knockdown cells (Supplementary Figure S3). Taken together, we conclude that cytoplasmic ATM is specifically required for β-TrCP- pIκBα complex formation, essential for rapid pIκBα proteolytic degradation in response to TNF treatment.

We next tested whether ATM participates in the IκBα·β-TrCP complex. β-TrCP was immunoprecipitated from CEs from TNF-exposed A549 cells and the bound ATM was detected by western blot. In the absence of stimulation, little ATM is associated with β-TrCP. However, TNF induced a rapid induction of ATM binding (Figure 2I). In this assay, p53, a protein known to bind ATM upon VP-16 addition (33), served as a positive control. The same experiment was also conducted in ATM-replete HeLa cells; similar results were obtained (Supplementary Figure S4).

Since different kinetics of IκBα degradation was observed in control shRNA and ATM shRNA transfected A549 cells, we further investigated the effects of ATM depletion on RelA nuclear translocation. For this experiment, NE from TNF stimulated control- or ATM shRNA-transfected A549 cells were prepared and assayed for RelA by western blot. In control shRNA-transfected A549 cells, a significant induction of RelA translocation was observed within 0.25 h, peaking within 0.5 h. By contrast, in ATM shRNA-transfected A549 cells, a decreased, but still obvious induction was observed (Figure 2J). The different kinetics of RelA nuclear translocation is consistent with the different kinetics of IκBα degradation in these cell lines (c.f. Figure 2B and D).

Taken together, these findings clearly suggest a novel role of cytoplasmic ATM to bind and recruit β-TrCP to pIκBα and explains the differences of kinetics in IκBα degradation, reduced β-TrCP·IκBα binding and stabilization of pIκBα in ATM-deficient cells.

### ATM is required for TNF-induced NF-κB phosphorylation on Ser ^276^ through the PKAc pathway

**Figure 3.**
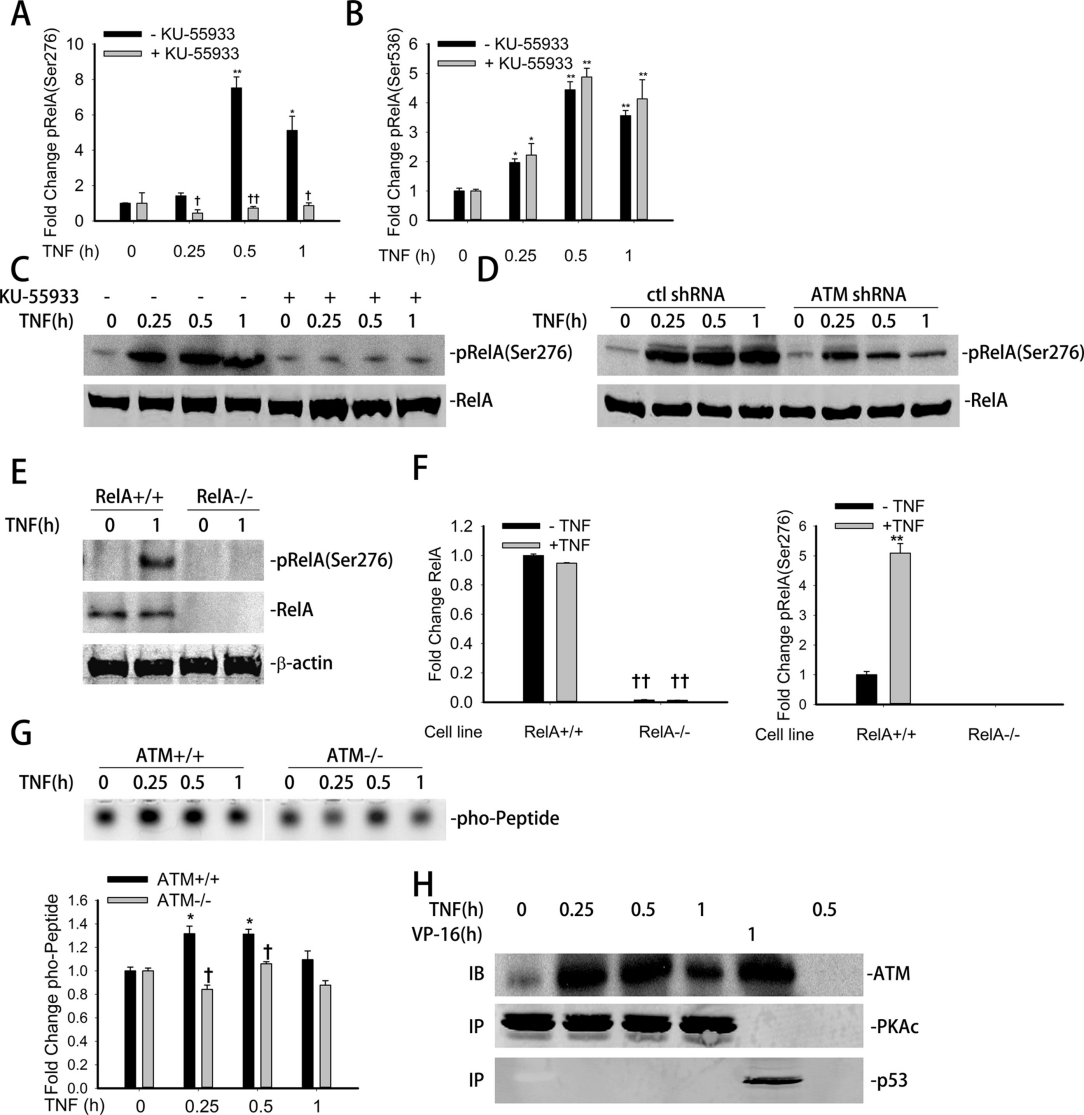
ATM is required for TNF-induced RelA Ser 276 phosphorylation through PKAc pathway. (**A**) A549 cells were treated with TNF (30 ng/ml) for the indicated time with or without the pretreatment of KU-55933 (10 μM, 1 h). Equal amounts of WCE were immunoprecipitated by pan anti-RelA Ab and subjected for SID-SRM-MS analysis using a phospho-Ser 276 RelA proteotypic peptide. All of the values are presented as the ratios of native to SIS peptides. (**B**) A549 cells were treated with TNF (30 ng/ml) for the indicated time with or without the pretreatment of KU-55933 (10 μM, 1 h). Equal amounts of WCE were immunoprecipitated by pan anti-RelA Ab and subjected for SID-SRM-MS analysis using a phospho-Ser 536 RelA proteotypic peptide. All of the values are presented as the ratios of native to SIS peptides. (**C**) A549 cells were treated with TNF (30 ng/ml) for the indicated time with or without the pretreatment of KU-55933 (10 μM, 1 h). Equal amounts of WCE were analyzed by WBs to detect the level of phospho-Ser 276 RelA. RelA were detected as internal control. (**D**) Control shRNA and ATM shRNA knockdown A549 cells were treated with TNF (30 ng/ml) for the indicated time. Equal amounts of WCE were analyzed by WBs to detect the level of phospho-Ser 276 RelA. RelA were detected as internal control. (**E**) RelA^+/+^ and RelA^−/−^ MEFs were treated with TNF (30 ng/ml) for 1 h. Equal amounts of WCE were assayed by western blot using anti-RelA and anti-phospho-Ser 276 RelA Abs. β-actin was measured as internal control (**F**) Equal amounts of WCE from TNF treated RelA^+/+^ and RelA^−/−^ MEFs were immunoprecipitated by anti-RelA Ab and then applied for SID-SRM-MS analysis to determine the RelA and phospho-Ser 276 RelA abundance. All of the values are presented as the ratios of native to SIS peptides. (**G**) Top panel. ATM^+/+^ and ATM^−/−^ MEFs were treated with TNF (30 ng/ml) for the indicated time. WCE were collected and PKAc activity was measured with PepTag nonradioactive assay reagents as described in the ‘Materials and Methods’ section. Bottom panel. Quantitation of the top panel. (**H**) A549 cells were treated with TNF (30 ng/ml) or VP16 (10 μM) for the indicated time. Equal amount of CE were immunoprecipitated by PKAc Ab (lane1–4), p53 Ab (lane 5) or rabbit preimmune serum (lane 6). Interacting ATM were measured by WBs.* Significantly different from TNF (0 h)-treated samples, *P* < 0.05;** Significantly different from TNF (0 h)-treated samples, *P* < 0.01; ^†^ Significantly different from ATM^+/+^ samples, *P* < 0.05; ^††^ Significantly different from ATM^+/+^ samples, *P* < 0.01.

Once released from IκBα inhibition, RelA is required for induction of downstream genes (5,34). In this process, RelA is phosphorylated at Ser residues 276 and/or 536 by distinct pathways (35,36). In particular, we have observed that TNF-induced phospho-Ser 276 RelA formation is ROS dependent, whereas phospho-Ser 536 RelA formation is ROS-independent (5). In order to examine whether ATM affects RelA phosphorylation on either Ser 276 or Ser 536, A549 cells were TNF-stimulated in the absence or presence of KU-55933 pretreatment. WCE were prepared and total RelA enriched by immunoprecipitation. Phospho-RelA was then quantitated in the immunoprecipitates by SID-SRM-MS using a proteotypic peptide corresponding to phospho-Ser 276 RelA (Materials and Methods). We observed that TNF induced an 8-fold induction of phospho-Ser 276 RelA formation after 0.5 h of treatment in control cells, whereas KU-55933 pretreatment significantly blocked phospho-Ser 276 RelA formation at all time points (Figure 3A). Quantitation of phospho-Ser 536 RelA formation showed that TNF induced an ∼4.5-fold induction of phospho-Ser 536 RelA in a manner that was not KU-55933 sensitive (Figure 3B). A western blot also showed the same result upon TNF stimulation with or without the pretreatment of KU-55933 (Figure 3C).

To further validate the role of ATM in RelA phosphorylation, we measured phospho-Ser 276 RelA levels in ATM knockdown cells. Either empty shRNA-transfected or ATM shRNA-transfected A549 cells were treated with TNF for indicated times. WCE were collected and RelA was immunoprecipitated. Phospho-Ser 276 RelA formation was quantitated by immunoblot. As expected, TNF treatment increased phospho-Ser 276 RelA formation in control shRNA transfectants starting from 0.25–1 h. However, TNF-induced phospho-Ser 276 RelA formation was significantly reduced in the ATM shRNA transfected cells (Figure 3D).

To ensure the antibody specificity for detection of phospho-Ser 276 RelA (37), WCE from control or TNF stimulated RelA^+/+^ and RelA^−/−^ MEFs were prepared and assayed by western blot. We noted a significant induction of phospho-Ser 276 RelA band in ATM^+/+^ MEFs while no signal was detected in ATM^−/−^ MEFs. The blot with anti-RelA Ab proved the knockout of RelA in ATM^−/−^ MEFs (Figure 3E).

Furthermore, we used RelA^+/+^ and RelA^−/−^ MEFs to validate the specificity of the IP-SRM assay for phospho-Ser 276 RelA. WCE from control or TNF stimulated RelA^+/+^ and RelA^−/−^ MEFs were prepared and immunoprecipitated by pan anti-RelA Ab. The abundanace of RelA and phospho-Ser 276 RelA were separately quantitated in the immunoprecipitates by SID-SRM-MS. Relative to that of RelA^+/+^ MEFs, very little RelA signal was detected (<0.1%) in RelA^−/−^ MEFs (Figure 3F, left panel). In RelA +/+ MEFs, phospho-Ser 276 RelA signal was detected and increased in response to TNF. Importantly, there was no signal of phospho-Ser 276 RelA detected in the RelA^−/−^ MEFs (Figure 3F, right panel). These data support the specificity of IP-SRM to measure phospho-Ser 276 RelA.

Earlier we reported that RelA Ser 276 phosphorylation is mediated by redundant ribosomal S6 kinases, including mitogen-stress related kinase-1 (MSK1) and catalytic subunit of active cAMP-dependent protein kinase A (PKAc) (5,38). Since PKAc is an ROS- sensitive kinase that mediates phospho-Ser 276 RelA formation, we investigated if PKAc activity is regulated by ATM. For this purpose, a PKAc kinase activity assay was performed in TNF stimulated ATM^+/+^ and ATM^−/−^ MEFs. In ATM^+/+^ MEFs, TNF induced a small, but significant increase in incorporation into the PKAc peptide at 0.25 and 0.5 h of stimulation (Figure 3G). When we examined the effect of ATM knockdown on MSK1 activation (phosphorylation at Ser 276), we did not observe any difference between wild type and ATM knockdown cells (data not shown). Together these data indicate that ATM is required for rapid induction of PKAc kinase activity in response to TNF treatment.

To investigate how ATM affects PKAc activity, we assessed whether ATM complexes with PKAc. PKAc was immunoprecipitated from control or TNF stimulated A549 cells and the immunoprecipitates were immunoblotted using anti-ATM Ab. In unstimulated cells, there was no detectable ATM. However, a strong ATM signal was observed 0.25 h after TNF stimulation peaking at 0.5 h (Figure 3H). p53, which is known to bind ATM upon VP-16 addition (33), serves as a positive control here. Overall, these data suggest that cytoplasmic ATM is essential for TNF-induced PKAc activation leading to RelA Ser 276 phosphorylation via a mechanism involving complex formation.

### ATM is required for a NF-κB-dependent immediate-early cytokine gene expression

**Figure 4.**
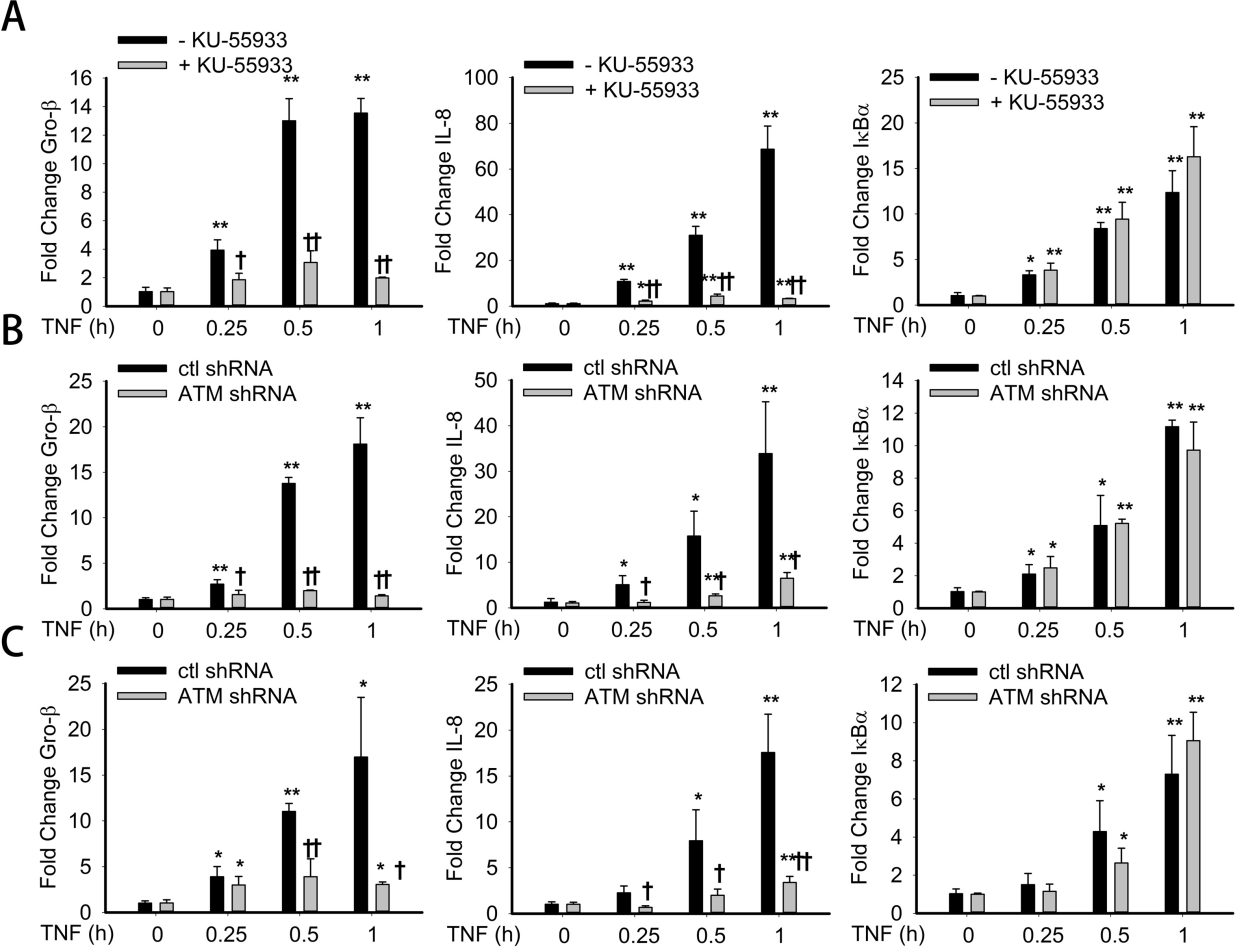
ATM in NF-κB-dependent immediate-early cytokine gene expression. (**A**). A549 cells were treated with TNF (30 ng/ml) for the indicated time with or without the pretreatment of KU-55933 (10 μM, 1 h). Total RNA was extracted. The mRNA levels of Gro-β, IκBα and IL-8 were measured. The results are expressed as fold change as compared with untreated cells after normalizing to internal controls, cyclophilin. Data represent the mean and STD of three independent experiments. (**B**). Control shRNA and ATM shRNA knockdown A549 cells were treated with TNF (30 ng/ml) for the indicated time. The experiment was performed as described for panel A. (**C**). Control shRNA and ATM shRNA knockdown HeLa cells were treated with TNF (30 ng/ml) for the indicated time. The experiment was performed as described for panel A. * Significantly different from TNF (0 h)-treated samples, *P* < 0.05;** Significantly different from TNF (0 h)-treated samples, *P* < 0.01; ^†^ Significantly different from ATM^+/+^ samples, *P* < 0.05; ^††^ Significantly different from ATM^+/+^ samples, *P* < 0.01.

Previously, we have reported that ROS-inducible RelA phosphorylation on Ser 276 is a post-translationally modified state required for the transcription of a subset of rapidly inducible NF-κB-dependent genes including the cytokines IL-8 and Gro-β, but not IκBα (5). Since ATM selectively affects RelA Ser 276 phosphorylation, we asked whether ATM is required for expression of these immediate-early genes. For this purpose, A549 cells were pre-treated in the absence or presence of KU-55933, TNF stimulated and expression of NF-κB-dependent gene expression performed by q-RT-PCR. In the absence of KU-55933, TNF induced a 15-fold increase in Gro-β expression at 0.5 and 1 h. This induction was significantly decreased by KU-55933 pretreatment (Figure 4A). A time dependent increase in IL-8 expression from 10-fold in 0.25 h to 70-fold after 1 h of TNF exposure was significantly blocked in cells pretreated with KU-55933 (Figure 4A). By contrast, IκBα, a gene whose expression is phospho-Ser 276 RelA independent (34), was induced to similar magnitude in control or KU-55933 pre-treated cells (Figure 4A). A similar effect on Gro-β and IL-8 expression was observed in ATM-shRNA transfected A549 cells, with IκBα being unaffected (Figure 4B). An identical pattern was seen in HeLa cells transfected with ATM shRNA (Figure 4C).

Previous studies have shown that the physiological level of TNFα in human plasma is much lower than the levels used in our experimental design to produce maximum responses (39,40). Therefore we next tested the requirement for ATM on NF-κB-dependent gene expression over a broad TNF dose-response range. For this experiment, control shRNA or ATM shRNA- transfected A549 cells were stimulated by escalating TNF doses (0.3, 3, 30 ng/ml) and IL-8 gene expression measured by q-RT-PCR. We observed a very sharp induction of IL-8 expression between 3 and 30 ng/ml, consistent with the known steep dose response of TNF signaling. However, at all doses of TNF, IL-8 expression was reduced in ATM shRNA transfected A549 cells (Supplementary Figure S5). Therefore, we conclude that ATM is required for TNF induced NF-κB-dependent gene expression over a broad physiological concentration range.

Overall, these studies demonstrated that ATM is required for the expression of a subset of phospho-Ser 276 RelA immediate-early genes.

### ATM is required for recruiting RelA and co-activators to immediate-early gene promoters

**Figure 5.**
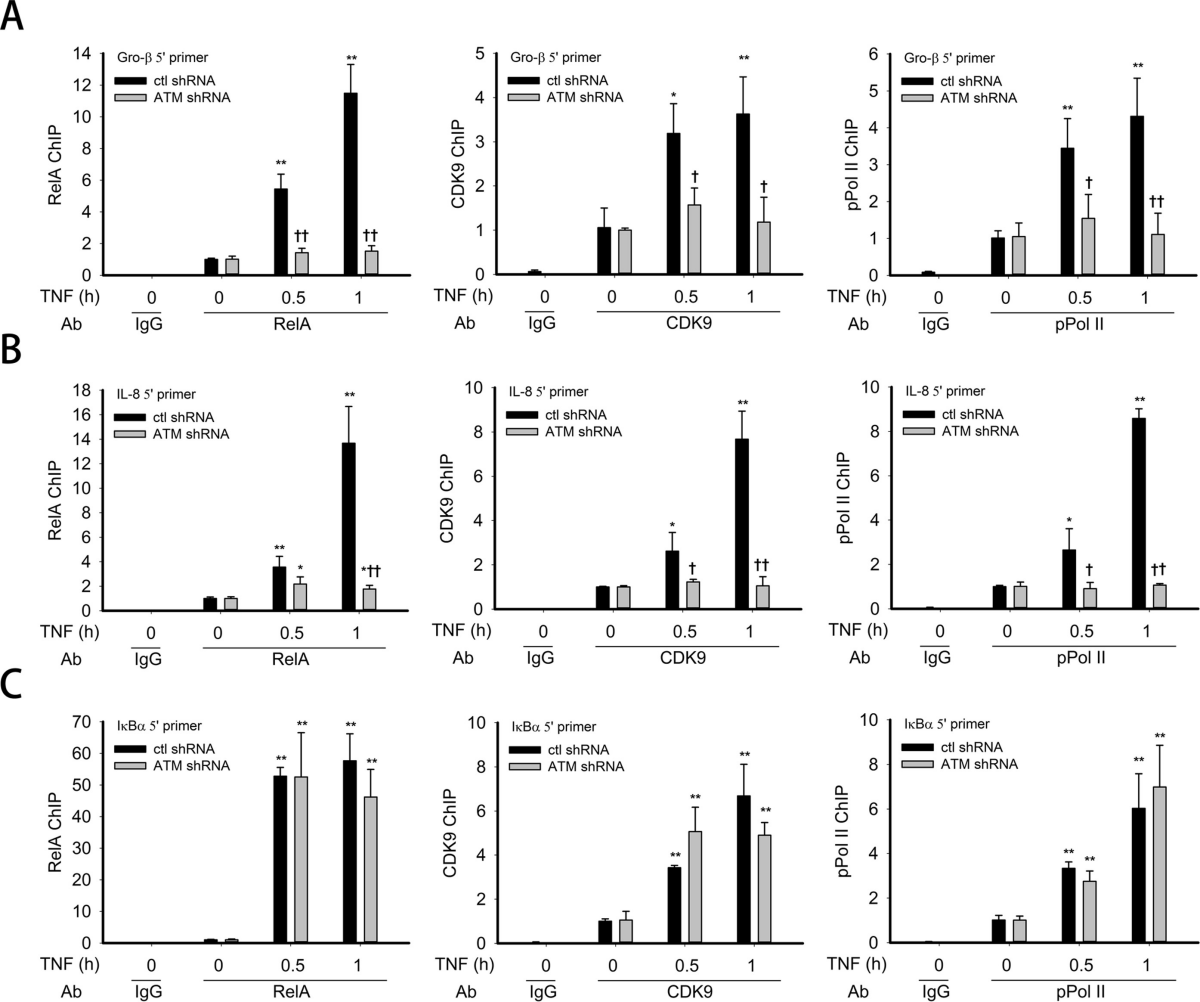
ATM in recruitment of RelA and co-activators to immediate-early gene promoters. (**A**) Control shRNA and ATM shRNA knockdown A549 cells were treated with TNF (30 ng/ml) for the indicated time. The corresponding chromatin was immunoprecipitated with anti-RelA, CDK9 and pPol II Abs. IgG was the negative control. q-RT-PCR was performed using the Gro-β 5′ primer set, and the fold change was calculated compared with unstimulated samples. (**B**) The experiment was performed as described for panel A. q-RT-PCR was performed using the IL-8 5′ primer set, and the fold change was calculated compared with unstimulated samples. (**C**) The experiment was performed as described for panel A. q-RT-PCR was performed using the IκBα 5′ primer set, and the fold change was calculated compared with unstimulated samples. * Significantly different from TNF (0 h)-treated samples, *P* < 0.05;** Significantly different from TNF (0 h)-treated samples, *P* < 0.01; ^†^ Significantly different from ATM^+/+^ samples, *P* < 0.05; ^††^ Significantly different from ATM^+/+^ samples, *P* < 0.01.

Earlier we have reported that phospho-Ser 276 RelA dependent gene expression is regulated via its interaction with cyclin-dependent kinase 9 (CDK9) resulting in RNA polymerase II (RNA Pol II) phosphorylation on Ser 2 of the heptad repeat in the C terminal domain (CTD), a biochemical event necessary for expression of Gro-β and IL-8 (but not IκBα) genes (34). We therefore investigated the role of ATM on targeting of CDK9 and RNA Pol II on IL-8, Gro-β and IκBα-containing chromatin by ChIP assay. For this purpose, A549 cells transfected with either control or ATM-specific shRNA were treated with TNF for the indicated times; chromatin was cross-linked and subjected to immunoprecitpitation with anti-RelA, CDK9 or phospho-Ser 2 Pol II Abs. Anti-rabbit IgG was used as a negative control. As expected, in control shRNA transfected A549 cells, TNF induced a 6-fold recruitment of RelA (after 0.5 h) and a 12-fold recruitment (after 1 h) on the Gro-β promoter. However, in cells transected with ATM-shRNA, RelA recruitment was significantly inhibited (less than 2-fold at these time points, Figure 5A). Similarly, TNF induction of CDK9 and phospho-Ser 2 RNA pol II recruitment was significantly induced in control shRNA transfectants and significantly inhibited in the ATM-shRNA transfectants (Figure 5A, middle and right panels).

A similar pattern of inhibition was observed for the IL-8 gene, whereas protein recruitment to the IκBα promoter was not affected (Figure 5B and C). These results provide a potential mechanism by which ATM-induced RelA posttranslational modifications regulate expression of a highly inducible subset of NF-κB-dependent genes.

## DISCUSSION

**Figure 6.**
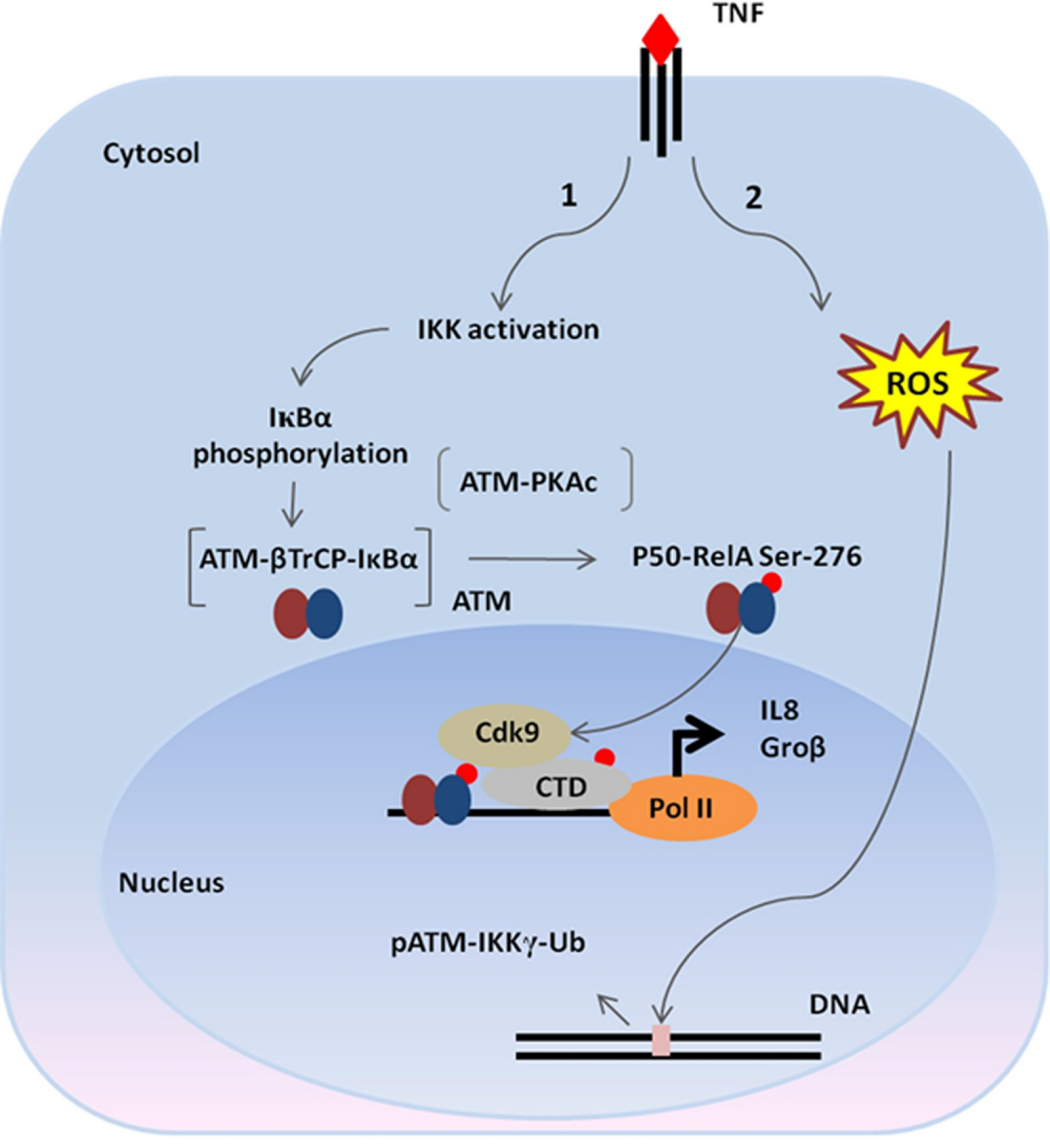
The model for the role of ATM in TNF pathway. Binding of TNF to the TNF receptor on the plasma membrane activates two parallel pathways: (1) IKK complex activation resulting in IκBα phosphorylation and (2) ROS generation leading to DNA double-strand breaks. The latter produces ATM activation and IKKγ-dependent nuclear export. Once in the cytosol, ATM facilitates two important steps of NF-κB activation pathway: rapid IκBα degradation by forming a complex with β-TrCP and RelA Ser 276 phosphorylation by interacting with PKAc.

In this study, we extend our previous discovery-based observation for a role of ATM in outside-in signaling in the innate immune response. Our data suggests that ATM is rapidly exported from the nucleus in response to TNF stimulation via an ROS-dependent pathway that affects IKKγ association with ubiquitin and complex formation with ATM. Activated ATM functions in regulating the NF-κB pathway at two key steps- one, affecting the rate of IκBα degradation, and the second, affecting RelA activation by Ser 276 phosphorylation (schematically diagrammed in Figure 6). In ATM knockdown cells, we have observed a decreased recruitment of the β-TrCP ubiquitin ligase to p-IκBα and its stabilization. In parallel, activated ATM forms a complex with PKAc, stimulating its activity and promoting phosphorylation of RelA Ser 276. This event is required for CDK9 recruitment and expression of immediate-early cytokine genes through phospho-Ser2 CTD Pol II formation and transcriptional elongation. Our studies suggest a coordinated signaling pathway involving ‘inside-out’, nuclear DNA-damage response pathways, and ‘outside-in’ plasma membrane located cytokine receptor signaling for full activation of the innate response.

Double-stranded DNA breaks are well-established to be the prototypical activating stimulus for ATM. ATM participates in the DSBs-repair response via phosphorylating and stabilizing the Nibrin/MRN complex on the DNA lesion (41). Our surprising findings from the Neutral Comet assays (Figure 1C) suggest that TNF induces dsDNA breaks and ATM activation over a time consistent with activation of the NF-κB signaling pathway. The mechanism for how TNF induces dsDNA breaks is not currently clear. However, compared to the level of DSBs induced by VP-16, TNF-induced DSBs are much less. An earlier study suggested that increased mitochondrial formation of ROS is responsible for inducing DNA damage after TNF exposure based on the finding that cell pretreatment with a reducing agent (dithiothreitol) or a ferric ion chelator (desferoxamine) prevented TNF-induced DNA damage (42). Another possibility is that DSB are produced by the process of base excision repair of oxidized DNA bases, generated by TNFα-induced oxidative stress. Recently, we have demonstrated that TNF-induces accumulation of 8-oxoG in DNA and the subsequent recruitment of 8-oxoguanine glycosylase 1 (OGG1), an 8-oxoG repair enzyme on the NF-κB-dependent gene promoter is essential for their mRNA expression (43). This intriguing finding provides a mechanism to explain how oxidative DNA damage potentially regulates transcriptional expression of NF-κB-dependent genes in TNF pathway. This suggests that the post-DNA lesion events could be an epigenetic mechanism to modulate gene expression. It will be of interest to examine the effects of OGG1 deficiency on TNF-induced DSBs.

Earlier work using robust exogenous ROS exposure (e.g. hydrogen peroxide at supraphysiological 250 μM concentrations) has concluded that oxidative stresses activate ATM. Here, nuclear oxidation leads to Cys-2991 oxidation of ATM and formation of disulfide dimers. Subsequently, ATM undergoes inter-molecular autophosphorylation to produce the characteristic Ser 1981 phosphorylation, a signature of activated ATM (13). Our finding that ATM undergoes serine autophosphorylation by physiological receptor-coupled pathways is novel to our knowledge. Mitochondrial- as well as NADPH-oxidase-derived ROS has been implicated in the TNF-induced cytotoxicity and apoptosis (42,44–48). Alternatively, TNF also induces ROS resulting in guanine, DCFDA oxidation and formation of carbonylated proteins (5). It is surprising that ATM autophosphorylation is antioxidant-resistant, and instead, ROS is required for nuclear export of the activated ATM by disrupting complex formation with IKKγ. These observations suggest that a separate nuclear signal stimulates ATM phosphorylation; this mechanism will require further exploration. Although, the ATM activation after genotoxic stress usually progresses with slower kinetic as compared to a rapid activation after TNF exposure. The broad difference in the kinetic of ATM pathway activation by these two exposures suggests that ATM could be warranted to perform unique cellular function in DSB repair and activation of the innate immunity pathway.

In studies of the prototypical genotoxic stress induced ATM-NF-κB activation pathway, Ser 1981 autophosphorylated ATM is required for phosphorylation of IKKγ, triggering a sumoylation-for-ubiquitination exchange on IKKγ. The ubiquitinated IKKγ-ATM complex is then exported from the nucleus (8). In a manner similar to that produced by genotoxic stimuli, our findings for the absence of detectable cytoplasmic ATM in IKKγ^−/−^ cells clearly suggest the vital role of IKKγ in TNF-induced ATM nuclear export (Figure 1F). Together with our co-immunoprecipitation results, these data suggest that TNF-induced ATM nuclear export also requires binding to nuclear IKKγ (Figure 1G). Interestingly, IKKγ association with polyubiquitin chains is ROS-dependent, where TNF induces the accumulation of nuclear ubiquitinated IKKγ, but this is blocked by free radical scavenger treatment (Figure 1H).

The ubiquitination of IKKγ is a critical regulatory step in controlling IKK activation. Here, IKKγ is well established to be covalently ubiquitinated both by Lys (K) 63-linked chemistry and linear ubiquitination by TRAF6 and the LUBAC E3 ligases, respectively (49). K63-linked modification of cytoplasmic IKKγ promotes oligomerization of the IKK-α and -β kinases, resulting in IKK autoactivation (50). Recent work has also shown that IKKγ binds to free polyubiquitin chains, triggering activation of the innate immune response. Our study extends the understanding of the effects of ROS on controlling IKKγ association or ubiquitination. One limitation of the trypsin-based SID-SRM assay is that it is unable to differentiate between free polyubiquitin chains, or covalent K63 linked chemistries. Our confirmatory experiments suggest that IKKγ may be binding to unconjugated polyubiquitin chains. However, we cannot exclude the possibility that the abundance of covalently linked Ub-IKKγ is below the limit of our assay detection. Further investigation is required on this issue. The nuclear IKKγ ubiquitin ligase(s) and ROS-dependent pathways controlling them will require further investigation.

In ATM^−/−^ MEFs as well as shRNA-mediated ATM knockdown cells, we observed cytoplasmic accumulation of phosphorylated IκBα and a delay in TNF-induced IκBα degradation (Figure 2B and D). IKK-mediated Ser phosphorylation of IκBα at NH2 terminal Ser-32 and Ser-36 is coupled with ubiquitination of Lys-20 and Lys-21 by the recruitment of SKP1-CUL-1-F-box protein (SCF) ubiquitin ligase in association with F-box protein β-TrCP. The SCF^β-TrCP^ E3-ubiquitin ligase is essential for IκBα degradation and subsequent p50•RelA release into the nucleus because ablation of β-TrCP results in accumulation of IκBs and complete inhibition of NF-κB activation (51–53). Our co-immunoprecpitation experiments showing: (i) that cytoplasmic ATM binds to β-TrCP in a TNF inducible manner; (ii) pIκBα is stabilized in TNF-stimulated ATM -deficient cells; and (iii) β-TrCP recruitment to pIκBα is decreased in ATM-deficient cells together suggest that ATM may serve as a scaffold for the formation of a viable E3-ligase-pIκBα complex (Figure 2H). This data also explains observations of reduced RelA nuclear translocation after TNF stimulus (Figure 2J).

Earlier, ATM was shown to regulate SCF^β-TrCP^ mediated-ubiquitination and degradation of mdm2 after DNA damage. Here, activated ATM directly phosphorylates casein kinase Iδ (CKIδ) resulting in nuclear translocation of phospho-CKIδ wherein it phosphorylates mdm2 thereby subjecting to subsequent ubiquitination by SCF^β-TrCP^ (54). Similar to ATM-mediated activation of CKIδ and mdm2 degradation, genotoxic stress induced ATM activates IKK leading to IκBα phosphorylation subjecting to ubiquitination and degradation (55). However, in the TNF response pathway, ATM seems to directly work at the level of E3 ubiquitin ligase recruitment to facilitate IκBα degradation.

The delayed clearance of IκBα in ATM deficient cells may be the result of other ancillary pathways responsible for IκBα degradation in the absence of ATM. In this context, we have reported a calpain-dependent degradation of IκBα after TNF exposure (56). Alternatively, a recent report suggests an IKK activation-independent pathway of IκBα degradation following ultraviolet irradiation, a potent ATM inducer. In this pathway, stimulus dependent nuclear accumulate IKKβ serves as an adaptor protein that constitutively interacts with β-TrCP through heterogeneous ribonucleoprotein-U (hnRNP-U) leading to IκBα ubiquitination and degradation (57). Studies to elucidate these pathways of IκBα degradation in the absence of ATM is under investigation in our laboratory.

Several reports have suggested that release of RelA from cytoplasmic inhibitors is necessary, but not sufficient to induce gene expression after TNF treatment (58–60). The post-translational modifications of RelA, especially phosphorylation at Ser 276 and Ser 536 are reported to have a significant effect on its ability to interact with transcriptional co-activators to initiate transcriptional initiation. Our findings that ablation of ATM reduces RelA Ser 276 phosphorylation, suggests a mechanism for how ROS mediates RelA Ser 276 phosphorylation in the TNF pathway (Figure 3A–D). Ser 276 phosphorylation is controlled in a stimulus-dependent manner by the ribosomal S6 kinases, PKAc and MSK-1 (5,38). PKAc is the major kinase activated by the TNF pathway (5). Our data for the first time shows that ATM is required for TNF-induced activation of PKAc, via its interaction with ATM in the cytoplasm (Figure 3G and H). Whether ATM directly phosphorylates and activates PKAc, or whether it functions in a scaffolding complex to promote its association with RelA, will require further investigation.

Earlier, we have reported that phospho-Ser 276 RelA binds to the transcriptional elongation complex containing CDK-9 and cyclin T1. The immediate-early genes require binding of phospho-Ser 276 RelA are activated by chromatin targeting of the CDK9/CCNT1 complex through transcriptional elongation (34). Consistent with these findings, we observed that knockdown of ATM specifically affects CDK9 and phospho-Ser 2 CTD Pol II recruitment and expression of IL-8 and Gro-β (Figure 4A and B). Interestingly, IκBα is RelA 276-independent and resistant to ATM deficiency (Figure 4C). Earlier studies have shown that IκBα gene expression is independent of phospho-Ser 276 RelA and CDK9 (6). Interestingly, another group has identified that in the VP-16 induced NF-κB activation, ATM binds to RelA directly and phosphorylates RelA on Ser 547, a post-translational modification that represses a subset of NF-κB-dependent genes (61). It is presently unknown to us whether TNF-induced NF-κB activation involves RelA phosphorylation on Ser 547. This question and the relationship between Ser 276 and Ser 547 phosphorylation will require further investigation.

Our findings for these multiple roles of ATM in regulating the innate pathway have implications for ataxia-telengiectasia (A-T), a rare autosomal recessive disorder. A-T patients lack a functional ATM protein and exhibit wide array of systemic defects including immunodeficiency, cerebral degeneration, progressive ataxia, premature aging, increased incidence of lymphoid tumorigenesis and type-2 diabetes (14). This phenotype suggests a vital role for ATM in the regulation of multiple cellular functions. More than half of A-T patients show humoral and cellular immunodeficiency that significantly contributes to morbidity and mortality in these subjects (62–64). One mechanism proposed to explain immunodeficiency has been immunoglobulin deficiency (both IgA and IgG2) but the role of ATM in innate immunity has not been investigated. Our studies suggest that ATM deficiency in A-T may be associated with primary defects in the innate pathway, affecting both the magnitude and kinetics of the NF-κB response. Defects in innate signaling in A-T could be a unifying explanation for global defects in adaptive immunity.

In conclusion, our current study provides a novel role of nuclear kinase ATM in TNF-induced NF-κB activation pathway. A rapidly activated ATM after TNF exposure regulates expression of a subset of genes by controlling two key steps in the pathway: (i) facilitating optimal IκBα degradation by forming a viable IκBα-β-TrCP-ubiquitin ligase complex and (ii) RelA Ser 276 phosphorylation by promoting R6K/PKAc association with RelA.

## SUPPLEMENTARY DATA

Supplementary Data are available at NAR Online.

SUPPLEMENTARY DATA
