# Supplementary material for: ATM regulates NF-κB-dependent immediate-early genes via RelA Ser 276 phosphorylation coupled to CDK9 promoter recruitment
Source: Nucleic Acids Res. 2014 Jun 21;42(13):8416–32. doi: 10.1093/nar/gku529 (PMC4117761; doi:10.1093/nar/gku529)
Supplement: SUPPLEMENTARY DATA [file supp_42_13_8416__index.html]

ATM regulates NF-κB-dependent immediate-early genes via RelA Ser 276 phosphorylation coupled to CDK9 promoter recruitment — SUPPLEMENTARY DATA 

# ATM regulates NF-κB-dependent immediate-early genes via RelA Ser 276 phosphorylation coupled to CDK9 promoter recruitment

## SUPPLEMENTARY DATA

**Files in this Data Supplement:**

- SUPPLEMENTARY DATA
