# Supplementary material for: ATM regulates NF-κB-dependent immediate-early genes via RelA Ser 276 phosphorylation coupled to CDK9 promoter recruitment
Source: Nucleic Acids Res. 2014 Jun 21;42(13):8416–32. doi: 10.1093/nar/gku529 (PMC4117761; doi:10.1093/nar/gku529)
Supplement: SUPPLEMENTARY DATA [file supp_gku529_nar-00379-x-2014-File008.pdf]

Supplementary Figure S1. Kinetics of piκbα in control shRNA and ATM shRNA transfected A549 cells. Control shRNA and ATM shRNA transfected A549 cells were treated with TNF (30 ng/ml) for the indicated time. Equal amount of CE were immunoprecipitated by anti-IκBα Ab. pIκBα levels were measured by Western blot.

Supplementary Figure S2. IκBα·β-TrCP complex formation in control and ATM silenced HeLa cells. Control shRNA and ATM shRNA transfected HeLa cells were treated with TNF (30 ng/ml) for the indicated time. Equal amount of CE were immunoprecipitated by anti-IκBα Ab. Interacting β-TrCP was measured by Western blot.

Supplementary Figure S3. β-TrCP and NF-κB2/p100 complex formation in control and ATM silenced A549cells. Control shRNA and ATM shRNA transfected A549 cells were treated with TNF (30 ng/ml) for the indicated time. Equal amount of CE were immunoprecipitated by NF-κB2/ p100 Ab. Interacting β-TrCP was measured by Western blot.

Supplementary Figure S4. ATM and β-TrCP complex formation after TNF stimulation in HeLa cells. HeLa cells were treated with TNF (30 ng/ml) or VP16 (10 μM) for the indicated time. Equal amount of CE were immunoprecipitated by β-TrCP Ab (lanes 1-4), p53 Ab (lane 5) or rabbit preimmune serum (lane 6). Interacting ATM was detected by Western blot.

Supplementary Figure S5. Dose titration of TNF induced IL-8 gene expression in ATM replete and knockdown cells. Control shRNA and ATM shRNA transfected A549 cells were treated with the indicated concentrations of TNF (1 h). Total RNA was extracted. The mRNA levels of IL-8 were measured. The results are expressed as fold change as compared with untreated cells after normalizing to cyclophilin as an internal control. * Significantly different from TNF (0 h)-treated samples, $p<0.05$;** Significantly different from TNF (0 h)-treated samples, $p<0.01$; † Significantly different from ATM WT samples, $p<0.05$; †† Significantly different from ATM WT samples, $p<0.01$.

Supplementary Figure S1

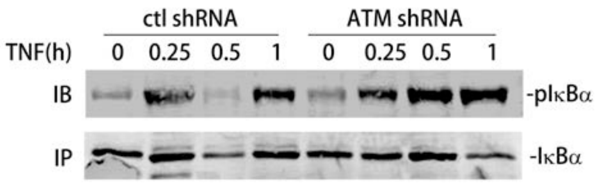

Supplementary Figure S2

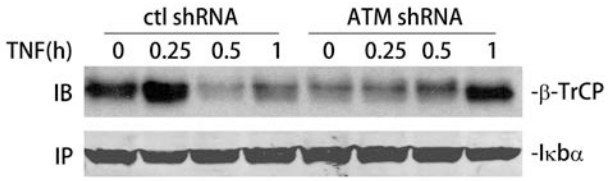

Supplementary Figure S3

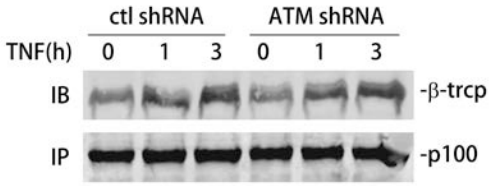

Supplementary Figure S4

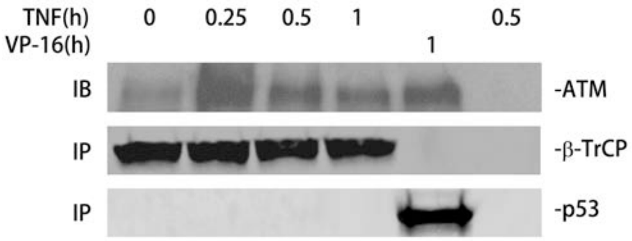

Supplementary Figure S5

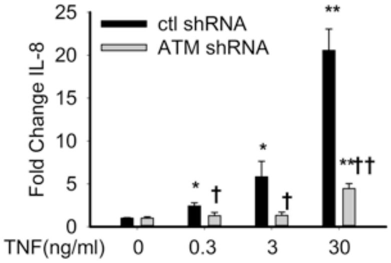

Fold Change IL-8
25
20
15
10
5
0
ctl shRNA
ATM shRNA
*
†
*
†
**
**††
TNF(ng/ml)
0
0.3
3
30
